# Danish first aid books compliance with the new evidence-based non-resuscitative first aid guidelines

**DOI:** 10.1186/s13049-018-0472-7

**Published:** 2018-01-10

**Authors:** Theo Walther Jensen, Thea Palsgaard Møller, Søren Viereck, Jens Roland, Thomas Egesborg Pedersen, Freddy K. Lippert

**Affiliations:** 1 Emergency Medical Services Copenhagen, University of Copenhagen, Telegrafvej 5, 2750 Copenhagen, Denmark; 2 Danish Resuscitation Council, c/o Emergency Medical Services, Telegrafvej 5, 2750 Copenhagen, Denmark; 3 Danish First Aid Council, c/o Dansk Folkehjælp, Brovejen 4, 4800 Nykøbing Falster, Denmark

**Keywords:** Implementation, First aid, Course material, Education, Resuscitation, Guidelines

## Abstract

**Background:**

The European Resuscitation Council (ERC) released new guidelines on resuscitation in 2015. For the first time, the guidelines included a separate chapter on first aid for laypersons. We analysed the current major Danish national first aid books to identify potential inconsistencies between the current books and the new evidence-based first aid guidelines.

**Methods:**

We identified first aid books from all the first aid courses offered by major Danish suppliers. Based on the new ERC first aid guidelines, we developed a checklist of 26 items within 16 different categories to assess the content; this checklist was adapted following the principle of mutually exclusive and collectively exhaustive questioning. To assess the agreement between four raters, Fleiss’ kappa test was used. Items that did not reach an acceptable kappa score were excluded.

**Results:**

We evaluated 10 first aid books used for first aid courses and published between 2009 and 2015. The content of the books complied with the new in 38% of the answers.

In 12 of the 26 items, there was less than 50% consistency. These items include proximal pressure points and elevation of extremities for the control of bleeding, use of cervical collars, treatment for an open chest wound, burn dressing, dental avulsion, passive leg raising, administration of bronchodilators, adrenaline, and aspirin.

**Conclusions:**

Danish course material showed significant inconsistencies with the new evidence-based first aid guidelines. The new knowledge from the evidence-based guidelines should be incorporated into revised and updated first aid course material.

**Electronic supplementary material:**

The online version of this article (10.1186/s13049-018-0472-7) contains supplementary material, which is available to authorized users.

## Background

First aid for laypersons was initially introduced in 1878 by the British army officers Peter Shepherd and Francis Duncan. Shepherd and Duncan’s curriculum included teaching laypersons how to use bandages and stretchers, as well as how to manage fractures, dislocations, burns, chemical exposure, drunkenness, seizures, fainting, and stroke [1]. Today, first aid is considered a part of daily life; consequently, there is a large commercial market for first aid courses. However, there is little scientific evidence to support current practices.

The International Liaison Committee on Resuscitation (ILCOR) task force, established in 2012, paved the way for the development of the first evidence-based first aid guidelines [2–4]. In October 2015, the European Resuscitation Council (ERC) released the new guidelines on resuscitation, which included for the first time first aid guidelines for laypersons. The ERC first aid guidelines cover 22 recommendations, each containing up to three items [5].

To change the clinical practice, some known barriers to implementation might need to be overcome [6, 7].

Regarding the new ERC first aid guidelines, implementation barriers include the lack of awareness of the new guidelines’ compliance with current practice. To assess the applicability and need for new training, it would beneficial to compare the content of existing national first aid books with the new guidelines; to date, no such systematic review has been reported in the literature. Consequently, we analysed the major current Danish national first aid books to identify compliance and potential inconsistencies with the new evidence-based ERC first aid guidelines. When considering the introduction of new items and updating the major existing first aid books, the benefits of introducing the item must be weighed against the disadvantages, and the effort and cost associated with introducing changes and updates to the material should be considered. The aim is to analyse whether the Danish non-resuscitative first aid books (and courses) are in compliance with the new ERC guidelines.

## Methods

In this study, we use the following definition of first aid from the ERC Guidelines for Resuscitation 2015 Section 9: “First Aid is defined as the helping behaviours and initial care provided for an acute illness or injury.” We examine the compliance with Section 9 isolated; hence, resuscitation with CPR and the use of AED are not included in the current analysis.

### Setting

Denmark has a population of 5.6 million, and approximately 300,000 (5%) participate in first aid courses each year [8]. In 2005, first aid education was introduced into the general curriculum in primary schools. In 2006, first aid courses have become a mandatory requirement to obtain a driver’s license. There are 10 widely used first aid books for laypersons that are distributed by the major first aid course suppliers in Denmark; these 10 books are structured in accordance with the International Red Cross and American Heart Association Committee for First Aid [9, 10]. The Danish first aid education is structured around principles of safety of the rescuer, call for help, first aid and assessing the need for life support [11].

### Materials

Our study was based on the books used in first aid courses in 2015, before publication of the new ERC first aid guidelines. We identified first aid books from all the major Danish suppliers of first aid courses via internet searches and direct contact with suppliers. We also consulted the Danish First Aid Council to ensure that all available books were identified.

### Development of the checklist

Based on the new ERC first aid guidelines, a checklist was developed and adapted to follow the principle of mutually exclusive and collectively exhaustive (MECE) questioning. The MECE principle contemplates the idea that effective mapping should answer only one essential part of that under examination at the time, that is, it should be distinct. Furthermore, all responses should fully address all the relevant parts of that are aimed to be mapped, that is, the responses should be exhaustive. Originally developed for the corporate world [12–14], the principle of mutually exclusive and collectively exhaustive questioning has been used in several medical research studies due to its ability to distinguish between differing items while ensuring that all items are enlightened [15–18].

The new ERC first aid guidelines include several different categories with distinct items that define actions in each category. The “Administration of oxygen” category was excluded because oxygen is considered a medical drug in Denmark; therefore, the administration of oxygen by laypersons is not allowed without a physician’s prescription. Additionally, the “Positioning of an unresponsive but breathing person” category was not included in our analysis because this category was not part of first aid courses at the time of the study. However, it is a part of basic life support courses and, hence, should be covered by BLS-AED courses. After these exclusions, our checklist included 18 categories comprising 28 items. Table 1 lists all the categories and items from the new guidelines that were included in our analysis.

**Table 1:** 2015 ERC first aid recommendations

Optimal position for a shock patient: *“Place individuals with shock into the supine (lying on back) position. Where there is no evidence of trauma use passive leg raising to provide a further transient (<7 min) improvement in vital signs.”*
Oxygen administration: *There are no direct indications for the use of supplemental oxygen by first aid providers.*
Bronchodilator administration: *“Assist individuals with asthma who are experiencing difficulty in breathing with their bronchodilator administration. First aid providers must be trained in the various methods of administering a bronchodilator.”*
Stroke recognition: *“Use a stroke assessment system to decrease the time to recognition and definitive treatment for individuals with suspected acute stroke. First Aid providers must be trained in the use of FAST (Face, Arm, Speech Tool) or CPSS (Cincinnati Pre-hospital Stroke Scale) to assist in the early recognition of stroke.”*
Administration of aspirin for chest pain: *“In the pre-hospital environment, administer 150–300 mg chew- able aspirin early to adults with chest pain due to suspected myocardial infarction (ACS/AMI).”*
Second dosage of adrenaline for anaphylaxis: *“Administer a second intramuscular dose of adrenaline to individuals in the pre-hospital environment with anaphylaxis that has not been relieved within 5 to 15 min by an initial intramuscular auto-injector dose of adrenaline.”*
Hypoglycaemia treatment: *“Treat conscious patients with symptomatic hypoglycaemia with glucose tablets equating to glucose 15–20g. If glucose tablets are not available, use other dietary forms of sugar.”*
Exertion-related dehydration and rehydration therapy: *“Use 3–8% oral carbohydrate–electrolyte beverages for rehydration of individuals with simple exercise-induced dehydration.”*
Exertion-related dehydration and rehydration therapy: *“Use 3–8% oral carbohydrate–electrolyte beverages for rehydration of individuals with simple exercise-induced dehydration.”*
Control of bleeding: *“Apply direct pressure, with or without a dressing, to control external bleeding where possible. Do not try to control major external bleeding by the use of proximal pressure points or elevation of an extremity. However it may be beneficial to apply localized cold therapy, with or without pressure, for minor or closed extremity bleeding.”*
Use of a tourniquet: *“ Use a tourniquet when direct wound pressure cannot control severe external bleeding in a limb. Training is required to ensure the safe and effective application of a tourniquet.”*
Straightening an angulated fracture *Do not straighten an angulated long bone fracture. Protect the injured limb by splinting the fracture. Realignment of fractures should only be undertaken by those specifically trained to perform this procedure.*
First aid treatment for an open chest wound: *“Leave an open chest wound exposed to freely communicate with the external environment without applying a dressing, or cover the wound with a non-occlusive dressing if necessary. Control localised bleeding with direct pressure.”*
Spinal motion restriction: *“The routine application of a cervical collar by a first aid provider is not recommended. In suspected cervical spine injury, manually support the head in position limiting angular movement until experienced healthcare provision is available.”*
Recognition of concussion: *“Although a concussion scoring system would greatly assist first aid providers in the recognition of concussion, there is no simple validated scoring system in use in current practice. An individual with a suspected concussion should be evaluated by a healthcare professional.”*
Cooling of burns: *“Actively cool thermal burns as soon as possible for a minimum of 10 min duration using water.”*
Burn dressings: *“Subsequent to cooling, burns should be dressed with a loose sterile dressing.”*
Dental avulsion: *“If a tooth cannot be immediately re-implanted, store it in Hank’s Balanced Salt Solution. If this is not available use propolis, egg white, coconut water, ricetral, whole milk, saline or phosphate-buffered saline (in order of preference) and refer the individual to a dentist as soon as possible.”*

The 2015 ERC first aid recommendations are displayed

*ERC*, Emergency Resuscitation Council

### Rating the books

We developed a manual, which covered all 18 categories of the new first aid guidelines, to use the checklist (Additional file 1). This manual contained the actual wording found in the guidelines, as well as the questions to be answered, with definitions of the answers “yes” or “no” for each question. Additionally, we provided an example for each question because some of the questions might be complex. Four individuals evaluated 3 of the 10 books independently, enabling us to assess the reliability of agreement among the four individuals. Fleiss’ kappa statistic was used to exclude items with inconsistent answering from further analysis; a value above 0.6 (substantial agreement) was considered acceptable agreement. Hereafter, one individual rated the remaining books.

## Results

**Fig. 1:**
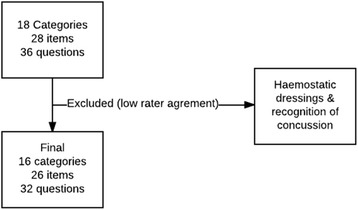
Book evaluation checklist The final checklist (after exclusions), which included 16 main categories and 26 total items, is displayed

Ten books published and used for teaching between 2009 and 2015 were evaluated using the checklist. The books varied from 68 to 170 pages in length. All books included a section on cardiopulmonary resuscitation, and 6 of the 10 books included a section on anatomy (Table 2). Items regarding haemostatic dressings and recognition of concussion were excluded from further analysis, due to low agreement among the reviewers (Kappa <0.6). Accordingly, the final checklist included 16 main categories comprising 26 items (Fig. 1).

**Table 2:** Overview of examined books

Book	Publication year	Pages	CPR instructions	Anatomy section
1	2009/2015	144	+	+
2	2011	128	+	+
3	2011	62	+	+
4	2011	127	+	+
5	2012	84	+	+
6	2012	109	+	–
7	2013	112	+	–
8	2015	170	+	+
9	2015	68	+	+
10	2015	142	+	+

*CPR* cardiopulmonary resuscitation

Overall, we found compliance with the new guidelines in 38% of the answers (122 of 320 answers in the checklist). Twelve items had more than 50% compliance with the guidelines, and 14 items had less than 50% compliance (Tables 3 and 4). Some items were not included in any of the books such as the administration of medicine (bronchodilators, adrenaline, and aspirin) and recommending against actions (proximal pressure points, elevation of extremities, the use of Stiffneck collars for suspected spinal injury). Other items not included in any of the books were the treatment of open chest wounds, burn dressings and the placement of teeth upon dental avulsion in fluids not customary to a Danish setting (Table 4).

**Table 3:** Items with more or less than 50% compliance^a^

Items with more than 50% compliance	Items with less than 50% compliance
Definition of shock as a condition with failing circulation.	Passive leg rise for shock victims with no sign of trauma.
Placement in the supine position for victims with shock.	Instructions on how to use inhalators or other bronchodilators.
Assist individuals with asthma with inhalators/bronchodilators.	Aspirin administration for chest pain.
System for the recognition of stroke.	Second dose of adrenaline for anaphylaxis.
Hypoglycaemia treatment.	Exertion-related dehydration and rehydration therapy.
Eye injury from chemical exposure.	Advised against the use of proximal pressure points to control bleeding.
Direct pressure to control external bleeding.	Advising against the elevation of extremities to control bleeding.
Immobilized of fractures in finding position.	Tourniquet use.
Dressing an open chest wound only with non-occlusive dressings.	Not straightening an angulated long bone fracture.
Manually support the head in position limiting movement. Placing avulsed teeth should in Milk.	Leaving open chest wound exposed to freely communicate with the external environment.
Contacting a dentist at dental avulsion.	Stopping localized bleeding on the chest with direct pressure.
	Spinal motion restriction
	Cooling of burns
	Avulsed teeth should be placed in; balanced salt solution, propolis, egg white, coconut water, phosphate buffered saline.

^a^ *Proportion of questions in the checklist with a “yes” answer rather than the answer “no”*

**Table 4:** Result of mapping compliance of Danish first aid books

Category	Item	Book a	Book b	Book c	Book d	Book e	Book f	Book g	Book h	Book i	Book j	+/-
1. Optimal position for a shock victim	1.a Is shock defined as a condition with failing circulation?	+	-	+	+	+	+	-	+	-	+	7/3
	1.b Is it mentioned that persons with shock should be placed in the supine position?	+	+	+	+	+	+	+	+	+	+	10/0
	1.c Is it mentioned that leg should be raised by “passive leg rise”, if there is no sign of trauma?	-	-	-	-	-	-	+	-	-	-	1/9
2. Bronchodilator administration	2.a Is it mentioned that one should assist individuals with asthma in taking their inhalators/bronchodilators if they have difficulty breathing?	+	+	+	+	-	-	+	+	+	+	8/2
	2.b Is there an explanation on how to use an inhalator or other bronchodilators?	-	-	-	-	-	-	-	-	-	-	0/10
3. Stroke recognition	3. Is there an explained system for the recognition of stroke?	+	+	+	+	+	-	+	+	-	+	8/2
4. Aspirin administration for chest pain	4. Does the book instruct in the use of acetylsalicylic acid/aspirin (ASA) for chest pain due to suspected myocardial infarction (ACS/AMI)?	-	-	-	-	-	-	-	-	-	-	0/10
5. Second dose of adrenaline for anaphylaxis	5. Is it recommended giving a second (repeated) intramuscular dose of adrenaline to persons with anaphylaxis?	-	-	-	-	-	-	-	-	-	-	0/10
6. Hypoglycaemia treatment	6. Does the book instruct readers to give glucose tablets of 15–20 g or equal glucose containing substance to persons with symptomatic hypoglycemia?	+	-	+	+	+	-	+	+	+	+	8/2
7. Exertion-related dehydration and rehydration therapy	7. Is it recommended to a se 3–8% oral carbohydrate–electrolyte beverages for rehydration of individuals with simple exercise-induced dehydration?	-	-	+	-	-	-	-	-	-	-	1/9
8. Eye injury from chemical exposure	8. Is it recommended irrigating eye injuries due to exposure to a chemical substance, with water?	+	-	+	+	+	+	+	+	+	+	9/1
9. Control of bleeding	9.a Is it recommended to apply direct pressure, (with or without a dressing) to control external bleeding?	+	+	+	+	+	+	+	+	+	+	10/0
	9.b Is it advised against the use of proximal pressure points, that is pressure applied centrally relative to the wound?	-	-	-	-	-	-	-	-	-	-	0/10
	9.c Is it advised against the elevation of extremities?	-	-	-	-	-	-	-	-	-	-	0/10
10. Use of a tourniquet	10. Does the book instruct readers to use a tourniquet when direct wound pressure cannot control severe external bleeding in a limb?	-	-	+	-	+	+	-	+	-	-	4/6
11. Straightening an angulated fracture	11.a Does the books state that on should not straighten an angulated long bone fracture?	-	-	-	-	-	-	+	-	-	-	1/9
	11.b Is the reader instructed to leave angulated fractures immobilized in the position in which it was found (possibly with a splint)?	+	+	+	+	+	+	+	+	+	+	10/0
12. First aid treatment for an open chest wound	12.a Is it clear that one should leave an open chest wound exposed to freely communicate with the external environment without applying a dressing, or cover the wound?	-	-	-	-	-	-	-	-	-	-	0/10
	12.b Is it described that one should stop localized bleeding on the chest with direct pressure?	+	-	-	-	-	-	-	-	-	-	1/9
	12.c Is it clear that dressing an open chest wound should only be done with a non-occlusive dressing (e.g. with a valve)?	+	+	+	+	+	-	+	+	+	-	8/2
13. Spinal motion restriction	13.a Does the book advise against the use of a cervical collar (e.g. Stiffneck)?	-	-	-	-	-	-	-	-	-	-	0/10
	13.b Is it recommended to manually support the head in position limiting angular movement?	+	-	+	+	+	+	+	+	+	+	9/1
14. Cooling of burns	14. Is it clear one should actively cool thermal burns as soon as possible and for a minimum of 10 minutes duration, using water?	-	-	-	+	+	+	+	-	-	-	4/6
15. Burn dressings	15. Is it clear that subsequent to cooling, burns should be dressed with a loose sterile dressing?	-	-	-	-	-	-	-	-	-	-	0/10
16. Dental avulsion	16.a Does the text state avulsed teeth should be placed in one of the following solutions if not able to immediately re-implanted?	+	-	-	-	-	-	+	-	-	+	3/7
	1). Balanced salt solution?	-	-	-	-	+	-	+	+	-	+	4/6
	2). Propolis?	-	-	-	-	-	-	-	-	-	-	0/10
	3). Eggwhite?	-	-	-	-	-	-	-	-	-	-	0/10
	4). Coconut water?	-	-	-	-	-	-	-	-	-	-	0/10
	5). Phosphate buffered Saline?	-	-	-	-	-	-	-	-	-	-	0/10
	6). Milk?	+	-	+	+	+	+	+	+	+	+	9/10
	16.b Does the book state that one should contact a dentist at dental avulsion?	+	+	+	+	+	-	-	+	-	+	7/3
Total compliant answers (number of “Yes” answer of 32 possible):		14	7	14	13	14	9	15	14	9	13	

The variation among the books was not large, with a median compliance of 13.5 (range 7–15) out of 32 questions. The most striking difference between books was between specific items such as the use of a tourniquet, general action in the case of dental avulsion, and a clear definition of (circulatory) shock. On a few items (passive leg raising, dehydration and rehydration therapy, and straightening an angulated fracture), only one book showed full compliance with the guidelines.

## Discussion

In this study, we analysed the current major Danish national first aid books and the lack of compliance and inconsistency with the new evidence-based ERC first aid guidelines. Of 26 first aid items investigated, 12 had more than 50% compliance with and 14 had less than 50% compliance with the new guidelines.

Observing all items and all books nationally, the Danish books were collectively in compliance with the guidelines of 38% just prior to the release of the new guidelines. In the following discussion, we wish to assess the applicability and feasibility of implementing the guidelines in a Danish context. We considered the costs and benefits of several implementing the recommendations behind items where there was an inconsistency between the new guidelines and current Danish books.

First, passive leg raising for shock victims is currently not included in the Danish books or practice. The ERC recommendation on passive leg raising is based on a scientific weak recommendation and only supported by low evidence. Nevertheless, training instructors on how to perform a passive leg rise would not require any further equipment. Furthermore, recognizing that it is a simple manoeuvre, the burden and expense of introducing passive leg raising to the books would be minimal, while the benefit to the patient is potentially high (studies indicate an increase in cardiac output and pulse pressure lasting up to 7 min during a passive leg rise [19–21]). Consequently, we argue for the implementation of passive leg raising guidelines into all Danish courses.

Second, when investigating inconstancy with recommendations on administering and dispensing medicine, we found a clear discrepancy between the guideline recommendations and current Danish materials. This is partially due to concerns among the Danish first aid suppliers that the administration of drugs should be reserved for healthcare professionals. When considering the potential cost of implementing the administration of drugs by laypersons, there are worries about the need for additional education and training, as well as fear of erroneous dose and administration. Furthermore, after a post-course retention period, erroneous administration by laypersons may have both legal and psychological consequences. Nevertheless, administering the victim’s own medication does not meet the same concerns. The use of some medications is backed by a high level of evidence (notably, aspirin) and may prove lifesaving if administered correctly. Therefore, we argue that some first aid personnel such as lifeguards and police could be trained to administer drugs, in the same way that first responders are trained to act in treating cardiac arrest.

Third, when considering dental avulsion, the cost of including additional solutions to the educational material for tooth placement seems low. Furthermore, no training is needed for a tooth placement-related manoeuvre or procedure. Although evidence is quite minimal and the recommendation is weak [5], there could be a benefit to including more potential solutions; a prioritized list could be reached with very low cost. In Scandinavia, egg whites are an available storage solution for dental avulsions and could be implemented at low cost. Consequently, we conclude that including guidelines on dental avulsion tooth placement-related procedures would be a painless addition to the current first aid books.

Fourth, when considering first aid care for burns, we found that the main component not already in compliance with the guidelines was that cooling a burn should take at least 10 min; this recommendation is supported by a low level of evidence, and the 10-min cut-off does not appear in any of the publications we examined [5, 22–24]. The implementation of cooling a burn for at least 10 min is almost costless; the associated wording simply needs to be added to the current books used. As a result, we believe these burn care instructions should be implemented into the current books and courses.

Fifth, using a tourniquet is not part of the Danish books and courses. Given that studies have indicated that personnel must be trained to effectively use tourniquets [25, 26], there is a training burden associated with tourniquet use. Retraining instructors on how to teach certain elements of first aid is expensive, but the benefits of someone learning how to use a tourniquet are potentially lifesaving [27–29]. Furthermore, tourniquets can be used by laypersons [30–32]. Therefore, we believe that tourniquet use should be added to the current Danish books and courses.

Sixth, instructions on leaving a chest wound open and controlling bleeding from open chest wounds were not standard in the Danish first aid books that we examined. The level of evidence for leaving a chest wound open and controlling bleeding from open chest wounds is low, but even valve dressings can create a pneumothorax, thereby emphasizing the importance of this issue [33]. We believe there is value to adding this element of chest wound care to the Danish books and courses.

Seventh, contrary to the ERC guideline recommendation that instructs not to elevate extremities to control bleeding, most first aid books we examined instructed first aid providers that they should elevate extremities. Changing the current approach from elevating to not elevating extremities would mean first aid instructors would have to be retrained. The benefit of not elevating extremities is somewhat uncertain because the recommendation is weak and is supported by a small amount of evidence. The two publications that support not elevating extremities are examining in-hospital cold compression [34, 35], which is just one of many situations where elevating extremities would need consideration. Due to a lack of evidence, ILCOR chose not to make a recommendation on whether to elevate extremities to control bleeding [36].

Eighth, none of the Danish books recommended using a loose sterile dressing for burns. Evidence for using loose sterile dressing is basically non-existent, so the outcome of implementing this first aid method is unknown [5]. If proven to be beneficial to patients, the cost of implementing the use of loose sterile dressings could be that instructors will need to be retrained. Additionally, finding sterile dressing is not always possible. Therefore, we argue that implementing the use of loose sterile dressing into the Danish books and courses could be somewhat challenging.

Finally, when considering the weak recommendation related to using carbohydrate-electrolyte beverages instead of other fluids to treat exertion-related dehydration and rehydration therapy, we believe laypersons may have a difficult time determining the ingredient content of specific beverages. Furthermore, the level of evidence supporting using carbohydrate-electrolyte beverages instead of other fluids is very low. In the twelve publications that do support this recommendation, there is a benefit to using carbohydrate-electrolyte beverages, but the benefit is not noticeably strong [5]. Consequently, we do not recommend adding the use of carbohydrate-electrolyte beverages in place of other fluids to the Danish first aid books.

### Strengths and limitations

A strength of our study was the use of the systematic and transparent principle of mutually exclusive and collectively exhaustive questioning, thus improving the reliability of our results and providing the possibility to re-test results, which merits comparison between countries.

However, there are some limitations that need to be addressed. First, we needed a manual to define the “yes” or “no” answers to the questions that were posed to ensure uniform coding between the individuals rating the books (Additional file 2). This manual was developed by the first author. However, the reliability of our results was strengthened by Fleiss’ kappa calculation and the subsequent exclusion of categories with low-level agreement. Second, the results could potentially lead people to believe that some books are superior compared to others. Rather, our analysis showed whether the content of the first aid books was consistent with the new evidence-based first aid guidelines. The first aid books were anonymized to avoid the commercial impact of potential findings and conclusions. Furthermore, table numbering was randomized to prevent the books from being recognized by suppliers and commercially influencing the results. This meant that we could not systematically report on the quality of the books.

### Future perspectives

The new evidence-based first aid guidelines have initiated a novel era in first aid care. Traditional first aid practices and teaching may not be in accordance with the current evidence-based guidelines. In some cases, we have recommendations on what to do or what not to do that are based on strong evidence; in these situations, the new evidence-based knowledge should be implemented as part of first aid courses and educational materials. In other cases, the recommendations on what to do or what not to do are based on weak evidence; in these situations, we need to consider the costs involved in changing the instructional material, retraining instructors, and overcoming implementation barriers. Thus, the current pedagogical trend is to provide a few, simple first aid messages to ensure proper implementation. Improving evidence in the first aid field is an ongoing process. Course materials and practice should be guided by evidence and international recommendations.

## Conclusion

We found a significant inconsistency between the new evidence-based ERC guidelines on non-resuscitative first aid and books from all major Danish suppliers of first aid courses released prior to these guidelines. The new ERC evidence-based guidelines should serve as a tool to standardize education material for first aid courses. Systematically analysing compliance between guidelines and national education material in first aid allows for better implementation, as well as local considerations regarding feasibility and costs.

## Additional files

Additional file 1: Questions in original language (Translated). (PDF 246 kb)

Additional file 2: Manual for answering questions (Translated). (PDF 537 kb)

## Abbreviations

ERC: European Resuscitation Council
ILCOR: International Liaison Committee on Resuscitation
MECE: Mutually Exclusive and Collectively Exhaustive
