# Supplementary material for: Danish first aid books compliance with the new evidence-based non-resuscitative first aid guidelines
Source: Scand J Trauma Resusc Emerg Med. 2018 Jan 10;26:7. doi: 10.1186/s13049-018-0472-7 (PMC5764019; doi:10.1186/s13049-018-0472-7)
Supplement: Supplementary file 1 — Questions in original language (Translated). (PDF 246 kb) [file 13049_2018_472_MOESM1_ESM.pdf]

Questions for analysis

This is an analysis of the contents of the Danish First Aid books and booklets in which we will examine whether the books and booklets comply with the ERC's section 9, in first aid.
In this analysis, no review of “positioning of a breathing but unresponsive victim” (1) is included. Oxygen administration for first aid (1) is not reviewed because it is not considered relevant to a layman in Denmark.
Some variations in the wording is expected in the books. If you believe that the meaning of the content is the same as the content a "yes" should be merited. If you are in doubt, it should be noted under the "Comments" field and subsequently discussed in the group.

The “Comments” field is ONLY intended for items where you are in doubt whether to answer "yes" or "no". If an item is NOT included in a book, you should mark "no".
If you have any doubts about how a question should be answered, you can look up the "*Manual for answering questions*”.

**1. Optimal position for a shock victim**	**Yes[x] / No[-]**	**Comments**
1.a Is shock defined as a condition with failing circulation?		
1.b Is it mentioned that persons with shock should be placed in the supine position?		
1.c Is it mentioned that leg should be raised by ”passive leg rise”, if there is no sign of trauma?		
**2. Bronchodilator administration**	**Yes[x] / No[-]**	**Comments**
2.a Is it mentioned that one should assist individuals with asthma in taking their inhalators/bronchodilators if they have difficulty breathing?		
2.b Is there an explanation on how to use an inhalator or other bronchodilators?		
**3. Stroke recognition**	**Yes[x] / No[-]**	**Comments**
3. Is there an explained system for the recognition of stroke?		
**4. Aspirin administration for chest pain**	**Yes[x] / No[-]**	**Comments**
4. Does the book instruct in the use of acetylsalicylic acid/aspirin (ASA) for chest pain due to suspected myocardial infarction (ACS/AMI)?		
**5. Second dose of adrenaline for anaphylaxis**	**Yes[x] / No[-]**	**Comments**
5. Is it recommended giving a second (repeated) intramuscular dose of adrenaline to persons with anaphylaxis?		
**6. Hypoglycaemia treatment**	**Yes[x] / No[-]**	**Comments**
6. Does the book instruct readers to give glucose tablets of 15–20 g or equal glucose containing substance to persons with symptomatic hypoglycaemia?		

**7. Exertion-related dehydration and rehydration therapy**	**Yes[x] / No[-]**	**Comments**
7. Is it recommended to a se 3–8% oral carbohydrate–electrolyte beverages for rehydration of individuals with simple exercise-induced dehydration?		
**8. Eye injury from chemical exposure**	**Yes[x] / No[-]**	**Comments**
8. Is it recommended irrigating eye injuries due to exposure to a chemical substance, with water?		
**9. Control of bleeding**	**Yes[x] / No[-]**	**Comments**
9.a Is it recommended to apply direct pressure, (with or without a dressing) to control external bleeding?		
9.b Is it advised against the use of proximal pressure points, that is pressure applied centrally relative to the wound?		
9.c Is it advised against the elevation of extremities?		
**10. Haemostatic dressing**	**Yes[x] / No[-]**	**Comments**
10.a Does the book instruct readers to use a haemostatic dressing when direct pressure cannot control severe external bleeding?		
10.b Does the book instruct user to apply direct pressure to control severe external bleeding?		
**11. Use of a tourniquet**	**Yes[x] / No[-]**	**Comments**
11. Does the book instruct readers to use a tourniquet when direct wound pressure cannot control severe external bleeding in a limb?		
**12. Straightening an angulated fracture**	**Yes[x] / No[-]**	**Comments**
12.a Does the books state that on should not straighten an angulated long bone fracture?		
12.b Is the reader instructed to leave angulated fractures immobilized in the position in which it was found (possibly with a splint)?		
**13. First aid treatment for an open chest wound**	**Yes[x] / No[-]**	**Comments**
13.a Is it clear that one should leave an open chest wound exposed to freely communicate with the external environment without applying a dressing, or cover the wound?		
13.b Is it described that one should stop localized bleeding on the chest with direct pressure?		
13.c Is it clear that dressing an open chest wound should only be done with a non-occlusive dressing (e.g. with a valve)?		
**13. Spinal motion restriction**	**Yes[x] / No[-]**	**Comments**
14.a Does the book advise against the use of a stiffneck?		
14.b Is it recommended to manually support the head in position limiting angular movement?		
**15. Recognition of concussion**	**Yes[x] / No[-]**	**Comments**
15. Is there an explained system for the recognition of concussion?		
**16. Cooling of burns**	**Yes[x] / No[-]**	**Comments**
16. Is it clear one should actively cool thermal burns as soon as possible and for a minimum of 10 min duration using water?		
**17. Burn dressings**	**Yes[x] / No[-]**	**Comments**
17. Is it clear that subsequent to cooling, burns should be dressed with a loose sterile dressing?		

18. Dental avulsion	Yes[x] / No[-]	Comments
18.a Does the text state avulsed teeth should be placed in one of the following solutions if not able to immediately re-implanted?<br>1). Balanced salt solution?<br>2). Propolis?<br>3). Eggwhite?<br>4). Coconut water?<br>5). Phosphate buffered Saline?<br>6). Milk?		
18.b Does the book state that one should contact a dentist at dental avulsion?		
