# Supplementary material for: Danish first aid books compliance with the new evidence-based non-resuscitative first aid guidelines
Source: Scand J Trauma Resusc Emerg Med. 2018 Jan 10;26:7. doi: 10.1186/s13049-018-0472-7 (PMC5764019; doi:10.1186/s13049-018-0472-7)
Supplement: Supplementary file 2 — Manual for answering questions (Translated). (PDF 537 kb) [file 13049_2018_472_MOESM2_ESM.pdf]

Manual for answering questions

Table of content

1. Optimal position for a shock victim 2
2. Bronchodilator administration 2
3. Stroke recognition 3
4. Aspirin administration for chest pain 3
5. Second dose of adrenaline for anaphylaxis 4
6. Hypoglycaemia treatment 4
7. Exertion-related dehydration and rehydration therapy 5
8. Eye injury from chemical exposure 5
9. Control of bleeding 5
10. Haemostatic dressing 6
11. Use of a tourniquet 7
12. Straightening an angulated fracture 7
13. First aid treatment for an open chest wound 7
14. Spinal motion restriction 8
15. Recognition of concussion 9
16. Cooling of burns 9
17. Burn dressings 9
18. Dental avulsion 10
References 10

1. Optimal position for a shock victim

ERC recommendation:
"*Place individuals with shock into the supine (lying on back) position. Where there is no evidence of trauma use passive leg raising to provide a further transient (<7 minutes) improvement in vital signs; the clinical significance of this transient improvement is uncertain.*"

1.a Is shock defined as a condition with failing circulation?

Requirements for answering "yes".	Requirements for answering "no".
To merit a "yes" answer the book has to offer a definition of shock as a failing/failed circulation. The wording is not essential if the substance is clear.<br>It is not wrong if the book state that shock is a failing peripheral circulation.<br>It is not wrong to state that shock can be a consequence of trauma or severe dehydration/hypovolemia	If the definition of shock is only described as a consequence of trauma or severe dehydration/hypovolemia without any further definition, you should answer "no".

1.b Is it mentioned that persons with shock should be placed in the supine position?

Requirements for answering "yes".	Requirements for answering "no".
The wording is not essential if the instruction leads to the victim being placed in the supine position (e.g. lay the person flat on the ground).	If the description appear to lead to placement of victim in any other position than the supine position, you should answer "no".

1.c Is it mentioned that leg should be raised by "passive leg rise", if there is no sign of trauma?

Requirements for answering "yes".	Requirements for answering "no".
The reader must be instructed to use passive leg raising for shock AND it should be clear that the shock should not be because of trauma.	If passive leg raising is not advised you should answer "no".

It is not considered wrong if the book states that passive leg raising can increase the circulatory function by approximately 7 minutes (e.g. maintain an acceptable blood pressure and stroke volume).

2. Bronchodilator administration

ERC recommendation:
"*Assist individuals with asthma who are experiencing difficulty in breathing with their bronchodilator administration. First aid providers must be trained in the various methods of administering a bronchodilator.* "

2.a Is it mentioned that one should assist individuals with asthma in taking their inhalators/bronchodilators if they have difficulty breathing?

Requirements for answering "yes".	Requirements for answering "no".
The wording is not essential. If the book describe the symptoms of asthma during an attack AND instruct in the use of inhalators/bronchodilators, you should answer "yes".	If administering inhalators/bronchodilators is not described, regardless of whether there is a description of asthma attacks, you should answer "no".

2.b Is there an explanation on how to use an inhalator or other bronchodilators?

Requirements for answering “yes”.	Requirements for answering “no”.
This is a broad question that does not require any essential wording for a “yes” answer. Answer “yes” if the essential substance includes any explanation of how to use inhalators or other means of administering bronchodilators.	If there is no mentioning on how to use inhalators or other means of administering bronchodilators, you should answer “no”.

3. Stroke recognition

ERC recommendation:
“*Use a stroke assessment system to decrease the time to recognition and definitive treatment for individuals with suspected acute stroke. First Aid providers must be trained in the use of FAST (Face, Arm, Speech Tool) or CPSS (Cincinnati Pre-hospital Stroke Scale) to assist in the early recognition of stroke*.“

3. Is there an explained system for the recognition of stroke?

Requirements for answering “yes”.	Requirements for answering “no”.
The specific method for recognition is not essential (e.g. FAST, CPSS or SSC). However, there should be some systemised approach to recognition of stroke including speech and paralysis.	If stroke is not covered or there is no system including speech and paralysis, you should answer ”no”.

Stroke may be labelled by other terms, if you are certain that stroke is covered (relevant in a Danish context).

4. Aspirin administration for chest pain

ERC recommendation:
“*In the pre-hospital environment, administer 150–300 mg chew- able aspirin early to adults with chest pain due to suspected myocardial infarction (ACS/AMI). There is a relatively low risk of complications particularly anaphylaxis and serious bleeding. Do not administer aspirin to adults with chest pain of unclear aetiology*.”

4. Does the book instruct in the use of acetylsalicylic acid/aspirin (ASA) for chest pain due to suspected myocardial infarction (ACS/AMI)?

Requirements for answering “yes”.	Requirements for answering “no”.
To answer “yes”, two elements must be covered in the book. Firstly, one drug containing acetylsalicylic acid (e.g. aspirin, Kodimagnyl, Treo, Hjerdyl) should be mentioned. Secondly, myocardial infarction should be mentioned or symptoms of myocardial infarction described (angina pectoris or generalized chest pain).	You should answer “no” if the book does not mention a name of any drug containing acetylsalicylic acid.

It is not considered relevant for the answer, whether the book mentions the dosage of acetylsalicylic acid. If the dosage of acetylsalicylic acid is mentioned, please note it in the comments if it is not between 150-300 mg.

5. Second dose of adrenaline for anaphylaxis

ERC recommendation:

"Administer a second intramuscular dose of adrenaline to individuals in the pre-hospital environment with anaphylaxis that has not been relieved within 5 to 15 min by an initial intramuscular auto-injector dose of adrenaline. A second intramuscular dose of adrenaline may also be required if symptoms re-occur."

5. Is it recommended giving a second (repeated) intramuscular dose of adrenaline to persons with anaphylaxis?

Requirements for answering "yes".	Requirements for answering "no".
To answer "yes", the book should cover administration of adrenalin with three essential parts. Firstly, it is essential that the book state that the adrenalin should be given as an injection. Secondly, it is essential that the book state that the injection should be given intra muscular.	If the three essential parts are not present in the book, you should answer "no".

Adrenalin can be labelled in a variety of ways (e.g. Epipen and epninephrine). The essential part in this item is that the substance contains adrenaline. If the dosage of adrenaline is mentioned, please note it in the comments if it is not between 0,3-0,5 mg per injection.

6. Hypoglycaemia treatment

ERC recommendation:

"Treat conscious patients with symptomatic hypoglycaemia with glucose tablets equating to glucose 15–20 g. If glucose tablets are not available, use other dietary forms of sugar."

6. Does the book instruct readers to give glucose tablets of 15–20 g or equal glucose containing substance to persons with symptomatic hypoglycemia?

Requirements for answering "yes".	Requirements for answering "no".
To answer "yes", the book should cover two essential elements. Firstly, symptoms of hypoglycaemia should be covered in some form. Relevant symptoms are tremor (shaking), palor (paleness), sudor (sweating), palpitation (e.g. flip-flopping or skipping a beat), hunger, cramps, mental disorder with irritation and aggression as well as fall in awareness. It is not important that all of these are mentioned as long as more than two of the mentioned symptoms are covered in the book. Secondly, the proposed treatment must include oral intake of a glucose containing substances. Neither which substance nor the concentration is essential.	If the two essential elements for a "yes" answer are not covered in the book, you should answer "no".

7. Exertion-related dehydration and rehydration therapy

ERC recommendation:

"Use 3–8% oral carbohydrate–electrolyte (CE) beverages for rehy-dration of individuals with simple exercise-induced dehydration. Alternative acceptable beverages for rehydration include water, 12% CE solution, coconut water, 2% milk, or tea with or without carbohydrate electrolyte solution added."

7. Is it recommended to use a 3–8% oral carbohydrate–electrolyte beverages for rehydration of individuals with simple exercise-induced dehydration?

Requirements for answering "yes".	Requirements for answering "no".
To answer "yes", the book should cover rehydration of individuals with exercise-induced dehydration with substances containing both carbohydrates and electrolytes. Electrolytes can also be labeled "salts". It is not essential for answering "yes" that the concentration of 3-8% is mentioned. It is considered correct if the book cover the item by mentioning CE-solutions, coconut water, milk or tea.	If the book does not instruct reader to give substances containing both carbohydrates and electrolytes of any sort, you should answer "no".

If concentrations are covered, you should note in the comments field if the concentration is not 3-8% carbohydrate–electrolytes.

8. Eye injury from chemical exposure

ERC recommendation:

"For an eye injury due to exposure to a chemical substance, take immediate action by irrigating the eye using continuous, large vol-umes of clean water. Refer the individual for emergency healthcare professional review."

8. Is it recommended irrigating eye injuries due to exposure to a chemical substance, with water?

Requirements for answering "yes".	Requirements for answering "no".
To answer "yes", the book should instruct readers to irrigate with water.	If the readers are instructed to use other substances than water, you should answer "no".

9. Control of bleeding

ERC recommendation:

"Apply direct pressure, with or without a dressing, to control external bleeding where possible. Do not try to control major external bleeding by the use of proximal pressure points or elevation of an extremity. However it may be beneficial to apply localised cold therapy, with or without pressure, for minor or closed extremity bleeding."

9.a Is it recommended to apply direct pressure, (with or without a dressing) to control external bleeding?

Requirements for answering "yes".	Requirements for answering "no".
To answer "yes", it essential that the book instruct the readers to apply direct pressure on the localized bleeding. The wording is not essential.	If the book does not cover either location (in the bleeding) or to apply pressure, you should answer "no".

9.b Is it advised against the use of proximal pressure points, that is pressure applied centrally relative to the wound?

Requirements for answering "yes".	Requirements for answering "no".
To answer "yes", the book should directly advise against proximal pressure points. It is not enough to not cover the item.	If the book does not advise against proximal pressure points, you should answer "no". If the item is not covered, you should also answer "no".

If proximal pressure points are recommended, please note this in the "comments" field.

9.c Is it advised against the elevation of extremities?

Requirements for answering "yes".	Requirements for answering "no".
To answer "yes", it should be explicitly advised against elevation of extremities in the book. It is not enough to not cover the item.	If the subject is not covered or there is no advice against the elevation of extremities, you should answer "no".

If proximal pressure points are recommended, please note this in the "comments" field.

10. Haemostatic dressing

ERC recommendation:

"Use a haemostatic dressing when direct pressure cannot control severe external bleeding or the wound is in a position where direct pressure is not possible. Training is required to ensure the safe and effective application of these dressings."

10.a Does the book instruct readers to use a haemostatic dressing when direct pressure cannot control severe external bleeding?

Requirements for answering "yes".	Requirements for answering "no".
To answer "yes", the book should clearly instruct to haemostatic dressings only when direct pressure cannot control a severe bleeding. It is not essential what haemostatic dressing is mentioned. It is not considered wrong if the book states that one should use a sterile dressing.	If it is not covered that rescues should try to control severe bleeding with direct pressure before applying a haemostatic dressing, you should answer "no".

10.b Does the book instruct user to apply pressure dressing?

Requirements for answering "yes".	Requirements for answering "no".
To answer "yes", the book should cover the item of applying pressure dressings. There is no one specific instructions that are essential. The essential part is that description results in a dressing that keep the dressing tight.	If there is no description of how to apply a dressing or if the application has no obvious pressure upon the wound, you should answer "no".

11. Use of a tourniquet

ERC recommendation:

"Use a tourniquet when direct wound pressure cannot control severe external bleeding in a limb. Training is required to ensure the safe and effective application of a tourniquet."

11. Does the book instruct readers to use a tourniquet when direct wound pressure cannot control severe external bleeding in a limb?

Requirements for answering "yes".	Requirements for answering "no".
To answer "yes", it is essential that the book instructs readers to apply a tourniquet ONLY when direct pressure cannot control a bleeding. It is not essential for a "yes" answer that the application of a tourniquet is described in a specific manner.	If the book does not cover tourniquet use or does not make it clear to the reader that one should first assess whether it is possible to apply direct pressure you should answer "no".

12. Straightening an angulated fracture

ERC's anbefaling:

"Do not straighten an angulated long bone fracture. Protect the injured limb by splinting the fracture. Realignment of fractures should only be undertaken by those specifically trained to perform this procedure."

12.a Does the book state that on should not straighten an angulated long bone fracture?

Requirements for answering "yes".	Requirements for answering "no".
To answer "yes", it should be explicitly advised against straightening an angulated fracture. It is not enough to not cover the item.	If the subject is not covered or there is no advice against straightening an angulated fracture, you should answer "no".

12.b Is the reader instructed to leave angulated fractures immobilized in the position in which it was found (possibly with a splint)?

Requirements for answering "yes".	Requirements for answering "no".
To answer "yes", it is essential that the book instruct readers to stabilize in the position in which it was encountered. The wording is not essential. If a splint or other aids are mentioned it is not considered wrong. However, it is not essential to include aids for a "yes" answer.	If the book does not instruct to stabilize in the position in which it was encountered ALL places in the book where the subject is covered, you should be answered "no".

13. First aid treatment for an open chest wound

ERC recommendation:

"Leave an open chest wound exposed to freely communicate with the external environment without applying a dressing, or cover the wound with a non-occlusive dressing if necessary. Control localised bleeding with direct pressure."

13.a Is it clear that one should leave an open chest wound exposed to freely communicate with the external environment without applying a dressing, or cover the wound?

Requirements for answering "yes".	Requirements for answering "no".
The book should state that one should not cover an open chest wound.	If the book gives the impression that one should make an occlusive dressing in any manner, you should answer "no".

12.b Is it described that one should stop localized bleeding on the chest with direct pressure?

Requirements for answering "yes".	Requirements for answering "no".
If the book explicitly state that only localised (e.g. visible) bleeding should be controlled by direct pressure, you should answer "yes".	If the book instruct rescuers to use a dressing to control bleeding or to control internal bleeding on the chest with direct pressure, you should answer "no".

12.c Is it clear that dressing an open chest wound should only be done with a non-occlusive dressing (e.g. with a valve)?

Requirements for answering "yes".	Requirements for answering "no".
To answer "yes", it should be clear that any proposed dressing is non-occlusive.<br>If the book states that you should leave the wound open without any dressing, this is also considered non-occlusive and you should answer "yes". If you find the description inconclusive (leaving the reader in doubt) you should not answer "yes".	If you find the description inconclusive (leaving the reader in doubt), you should answer "no".

14. Spinal motion restriction

ERC recommendation:

"The routine application of a cervical collar by a first aid provider is not recommended. In suspected cervical spine injury, manually support the head in position limiting angular movement until experienced healthcare provision is available."

14.a Does the book advise against the use of a Stifneck?

Requirements for answering "yes".	Requirements for answering "no".
To answer "yes", the book must explicitly advise against the use of cervical collars (e.g. Stifnecks),	If the book does not mention a cervical collar of any kind OR advise the use of cervical collars, you should answer "no".

14.b Is it recommended to manually support the head in position limiting angular movement?

Requirements for answering "yes".	Requirements for answering "no".
You should answer "yes" if the book instructs readers to support the head manually (without any aids).	If the book does not instruct readers to support the head or ONLY instruct in the use of aids (e.g. cervical collar) you should answer "no".

15. Recognition of concussion

ERC recommendation:

"Although a concussion scoring system would greatly assist first aid providers in the recognition of concussion, there is no simple validated scoring system in use in current practice. An individual with a suspected concussion should be evaluated by a healthcare professional."

15. Is there an explained system for the recognition of concussion?

Requirements for answering "yes".	Requirements for answering "no".
Not one specific system is essential, neither is the consequences for first aid. It is only essential to answer "yes" that a systematic approach to recognition concussion is present in the book.	If there is no system/tool for recognition of concussion, you should answer "no".

Concussion can also be labelled head trauma or head injury etc.

16. Cooling of burns

ERC recommendation:

"Actively cool thermal burns as soon as possible for a minimum of 10 min duration using water."

16. Is it clear one should actively cool thermal burns as soon as possible and for a minimum of 10 min duration using water?

Requirements for answering "yes".	Requirements for answering "no".
The instruction needs to include both irrigation with water AND the duration of minimum 10 minutes.	If the book does not include instruction of BOTH water irrigation and duration above 10 minutes, you should answer "no".

17. Burn dressings

ERC recommendation:

"Subsequent to cooling, burns should be dressed with a loose sterile dressing."

17. Is it clear that subsequent to cooling, burns should be dressed with a loose sterile dressing?

Requirements for answering "yes".	Requirements for answering "no".
To answer "yes", the book must mention that the dressing should be loose and sterile.	If burn dressings are covered either without a description matching loose or sterile dressing, you should answer "no".

18. Dental avulsion

ERC recommendation:

"If a tooth cannot be immediately re-implanted, store it in Hank's Balanced Salt Solution. If this is not available use propolis, egg white, coconut water, ricetral, whole milk, saline or Phosphate Buffered Saline (in order of preference) and refer the individual to a dentist as soon as possible."

18.a Does the text state avulsed teeth should be placed in one of the following solutions if not able to immediately re-implanted?

1). Balanced salt solution?

2). Propolis?

3). Eggwhite?

4). Coconut water?

5). Phosphate buffered Saline?

6). Milk?

Requirements for answering "yes".	Requirements for answering "no".
If just one of the medias for storage is mentioned, you should answer "yes". In the "comments" field it should be noted which medias are covered. It is not essential to answer "yes" that the book recommends re-implantation. However, it should be noted in the comments field if re-implantation is not recommended.	If none of the medias are covered in the book, you should answer "no".

18.b Does the book state that one should contact a dentist at dental avulsion?

Requirements for answering "yes".	Requirements for answering "no".
It is essential that the book include the word "dentist".	If the book does not include the word "dentist", you should answer "no".

References

1. Zideman DA, De Buck ED, Singletary EM, Cassan P, Chalkias AF, Evans TR, et al. European Resuscitation Council Guidelines for Resuscitation 2015 Section 9. First aid. Resuscitation. 2015;95:278-87.
